# Behavioral and multimodal neuroimaging evidence for a deficit in brain timing networks in stuttering: a hypothesis and theory

**DOI:** 10.3389/fnhum.2014.00467

**Published:** 2014-06-25

**Authors:** Andrew C. Etchell, Blake W. Johnson, Paul F. Sowman

**Affiliations:** ^1^Department of Cognitive Science, ARC Centre of Excellence in Cognition and its Disorders, Macquarie UniversitySydney, NSW, Australia; ^2^Department of Cognitive Science, Perception in Action Research Centre, Macquarie UniversitySydney, NSW, Australia

**Keywords:** stuttering, rhythm, tapping, speech, basal ganglia, cerebellum, timing

## Abstract

The fluent production of speech requires accurately timed movements. In this article, we propose that a deficit in brain timing networks is one of the core neurophysiological deficits in stuttering. We first discuss the experimental evidence supporting the involvement of the basal ganglia and supplementary motor area (SMA) in stuttering and the involvement of the cerebellum as a possible mechanism for compensating for the neural deficits that underlie stuttering. Next, we outline the involvement of the right inferior frontal gyrus (IFG) as another putative compensatory locus in stuttering and suggest a role for this structure in an expanded core timing-network. Subsequently, we review behavioral studies of timing in people who stutter and examine their behavioral performance as compared to people who do not stutter. Finally, we highlight challenges to existing research and provide avenues for future research with specific hypotheses.

## Theories of stuttering

According to the World Health Organisation (2010, para. F98.5), stuttering is “speech that is characterized by the frequent repetitions or prolongation of sounds or syllables or words, or by frequent hesitations or pauses that disrupt the rhythmic flow of speech.” Repetitions typically consist of a repetition of part of a word, a whole word or a phrase (e.g., re… re… re… repetitions). Prolongations consist of a lengthening of the sounds within a word (e.g., prrrrrrrolongations). Complete interruption to the flow of speech, known as “blocking” is also a common symptom of stuttering. Blocks are where there is a length of time where no form of speech is produced either within words [e.g., block-(pause)-ing] or between words. In most cases, stuttering emerges between 2 and 5 years of age, around the time children start preschool. Stuttering has a prevalence of around 5% in early childhood but due to the fact that many children recover spontaneously, the prevalence across the general population is closer to 1% (Yairi and Ambrose, 2013). This percentage of stutterers who do not recover generally experience poorer social, emotional and mental health (Craig et al., 2009; Iverach et al., 2009) and elicit negative reactions from others (Langevin et al., 2010). Stuttering is also associated with secondary or associated signs that include facial grimaces, forced effort and eye-blinks (Conture and Kelly, 1991; Riva-Posse et al., 2008). These secondary signs further impair the ability to communicate effectively and exacerbate the problems that result from the primary symptoms. Importantly, such secondary signs imply that stuttering is not solely confined to the domain of speech but rather a disorder of motor control that manifests primarily in the domain of speech because of the extreme timing and sequencing demands required for that function. Moreover, while difficult, it is not impossible to detect differences related to stuttering in the manual domain (e.g., Max et al., 2003; Ambrose, 2004).

Packman (2012) argues that the necessary condition for stuttering, i.e., the one thing each person who stutters must possess, is a neural anomaly that weakens the integrity of the speech motor system. In this weakened state, the speech motor system is rendered more susceptible to breakdown when various features of the spoken language place increasing demand on the system (Packman, 2012). The point at which stuttering is triggered is modulated according to individual and environmental factors such as levels of physiological arousal. Here we take the view that the necessary condition for stuttering (which unless otherwise specified is used to refer specifically to developmental stuttering) is the presence of a neural anomaly in timing.

The following account proposes the hypothesis that the core disorder of stuttering is a deficit in brain timing-networks. This article is not an exhaustive review of the literature on stuttering or the arguments surrounding the cause of the disorder, but rather a hypothesis as to one of the possible causes of stuttering. The proposal that timing is important for speech (see Lashley, 1951; Martin, 1972; Strait et al., 2011) and even speech disorders like specific language impairment (Tallal et al., 1993) dyslexia (Goswami, 2011) or indeed stuttering (Alm, 2004, 2010) is not new. In the later case, the idea that stuttering relates to a deficit of timing follows from the observation that regular external stimulation temporarily alleviates stuttering (see for a revision, Alm, 2004; Snyder et al., 2009). The novel aspect of this article is that it expands on previous research suggesting that dysfunction within a brain network that supports internal timing [comprised of the basal ganglia (BG) and the supplementary motor area (SMA)] is causing stuttering and that a secondary system which utilizes external timing cues to sequence movements [comprised of the cerebellum (CB), the premotor cortex (PMC) and the right inferior frontal gyrus (IFG)] is compensating for stuttering. Specifically, we propose that an internal timing network (ITN), largely equivalent to the “medial system” proposed by Goldberg (1985) is involved in internally timed movement (movement performed in the absence of external timing cues) and is causally related to stuttering. We further propose that an external timing network (ETN), largely equivalent to the “lateral system” proposed by Goldberg (1985), with the addition of the right IFG, is involved in externally timed movement (movement performed in the presence of external timing cues) and provides a substrate for timing compensation in stuttering. Importantly, we are not suggesting that neural deficits in structures underlying timing is the sole cause of stuttering, but rather one of many possible deficits that could lead to stuttering. In this section, we first present multimodal neuroimaging evidence for the possible causal involvement of ITN in stuttering before moving on to discuss putative compensatory roles of the ETN.

There is ongoing debate as to whether some brain regions are specifically dedicated to processing time or whether the capacity to process time is intrinsic to each region of the brain directly through the activation of sensory processes (for review see Ivry and Schlerf, 2008). There already exist reviews outlining the cognitive and neural architecture proposed for how we represent a sense of time (e.g., Buhusi and Meck, 2005), how different sensory networks interact with core timing networks across different tasks (e.g., Merchant et al., 2013) as well as evidence for common timing mechanisms across manual and oral movements (e.g., Franz et al., 1992). While the questions of how and where time is processed in the brain are of considerable practical and theoretical interest, such a discussion is outside the scope of this article. Here we argue that the ETN is primarily active when an individual is timing their movement to an external rhythm and that it is particularly active during early exposure to rhythm or when the rhythm is difficult and is not easily internalized. In contrast to this, the ITN is primarily active when an individual is making rhythmic motor movements that are not specifically timed to an external stimulus. Importantly, the two systems can be active simultaneously such as when an individual is pacing their movements to an external stimulus and is internalizing that rhythm. Practically, this means that results of functional magnetic resonance imaging (fMRI) studies may show no difference in brain activation between conditions that supposedly bias internally or externally-timed movements; however, disruption of these systems via inhibitory transcranial magnetic stimulation (TMS) should yield selective interference in behavioral performance. What follows is a brief overview of studies supporting a dissociation between the ITN and the ETN in timing tasks.

There is strong support for the involvement of the ITN during timing tasks from a number of fMRI, magnetoencephalography (MEG), lesion and TMS studies. For example, a recent fMRI study has found that the BG and the SMA tend to be active when movements are internally as opposed to being externally timed (Coull et al., 2013). Similarly, it has been shown using finger tapping tasks, that the BG and the SMA are active during the continuation phase (no external pacing stimulus, hence an internally-timed process) but not the synchronization phase (with external pacing, hence externally-timed) of the task (Rao et al., 1997). In particular, the BG are more active during the performance or tracking of simple rhythms, i.e., those that are easier to internalize, compared to complex rhythms (Grahn and Rowe, 2009, 2013; Geiser et al., 2012). The fact that fMRI studies show an overlap of neural activity during synchronization and continuation tapping (e.g., Jäncke et al., 2000; Jantzen et al., 2004) provides little support for a functional distinction between brain networks supporting internal and external timing; however, evidence from lesion and TMS does support such a dissociation between the INT and the ETN and their respective functions. Studies show that individuals with bilateral lesions to the BG perform poorly on the continuation phase of the finger-tapping task (Coslett et al., 2010) and are also poor at adjusting to accelerations and decelerations in tempo (Schwartze et al., 2011). Disruption of the SMA by inhibitory TMS impairs accuracy of continuation tapping whilst leaving the accuracy of synchronization tapping intact (Halsband et al., 1993).

There is also evidence for the involvement of CB and the PMC in the ETN. Inhibitory TMS of the CB has been shown to disrupt synchronization to auditory (Del Olmo et al., 2007) and visual pacing (Theoret et al., 2001; Koch et al., 2007). This disruption appears to be selective because lesions to the CB do not affect performance during the continuation phase of the finger-tapping task (Spencer et al., 2003). Likewise, a number of studies show that inhibitory TMS of the left PMC disrupts the synchronization tapping (Pollok et al., 2008; Bijsterbosch et al., 2011) and that this effect is specific to external pacing, as no effect of TMS is observed on continuation tapping (Del Olmo et al., 2007) or when tapping in the presence of, but not in time with, a scrambled beat (Kornysheva and Schubotz, 2011). Taken together, there indeed appears to be a functional dissociation of the ITN and the ETN in healthy adults. We now turn to neuroimaging studies to demonstrate how these systems are impaired in people who stutter.

## Neuroimaging studies of the internal timing network in PWS

A number of neuroimaging studies implicate the BG or components thereof in the etiology of stuttering. For example, when comparing the fluent and dysfluent speech of people who stutter (PWS) to people who do not stutter (PWDS), Wu et al. (1995) found that PWS exhibited less activity in the caudate during both dysfluent speech and fluent speech. This lowered activity was suggested to be a trait marker for stuttering. The BG has also been related to the most typical symptoms of stuttering at an individual level (Jiang et al., 2012). These authors elicited stuttering during a sentence completion task and classified repetitions, pauses and prolongations as being either least typical or most typical of stuttering based on patterns of haemodynamic responses. Jiang et al. (2012) found that one of the activation patterns contributing to this separation of most and least typical symptoms was a reduction in BG activation. Although the aforementioned studies provide a correlative link between the putative ITN and stuttering, they do not unambiguously support the notion that the ITN *causes* stuttering. Because those studies were conducted mainly in adults, and stuttering is a disorder that appears in childhood, it can therefore be hard to determine whether anomalous BG activations observed in PWS are related to the cause of stuttering or are compensations for it.

In contrast, structural and functional abnormalities in children who stutter (CWS) are likely to be more indicative of the causative agents in stuttering because children have not had as much time to adapt to stuttering as adults. Chang and Zhu (2013), examined functional connectivity in CWS and children who do not stutter (CWDS) aged 3–9 and found reduced levels of connectivity between the putamen and the SMA, superior temporal gyrus (STG) and CB and similarly between the SMA and the putamen, STG and CB. Chang and Zhu (2013) concluded that CWS exhibited reduced activity in areas responsible for self-paced movement as compared to CWDS. Similarly, a recent voxel based morphometry (VBM) study conducted in CWS, found less gray matter volume in the bilateral inferior frontal gyri and the left putamen but more gray matter volume in the right rolandic operculum and the right STG relative to CWDS (Beal et al., 2013). In another study, Foundas et al. (2013) measured the volume of the caudate in right-handed boys who stutter and compared them to right-handed boys who did not stutter. They found that male CWS exhibited significantly less volume in the right caudate as compared to male CWDS. These studies suggest that even at a very young age, CWS exhibit abnormalities in structure and connectivity in the ITN. A recent MEG study examined lateralization of brain functions in preschool CWS and CWDS during a picture-naming task (Sowman et al., 2014). These authors found that speech was strongly left lateralized in both groups. Although not explicitly focusing on the ITN, this study demonstrates that much of the abnormal activation observed in the cortical right hemisphere in adults is the result of years of compensation for stuttering rather than being causally related to it. Moreover, that there were no differences between CWS and CWDS in cortical activations further hints at the possibility that stuttering is caused by deficiencies in subcortical regions. Overall, these studies provide strong support for viewing stuttering as a disorder of the BG. Since the BG seems responsible for internal timing of movement, they provide indirect support that stuttering is a disorder of internally timed movement.

To implicate the ITN in stuttering, structural or functional abnormalities should be evident in these structures in both children and adults who stutter and the neural deficit necessary to cause stuttering should be present irrespective of whether or not a subject is performing a task. Ingham et al. (2012) examined speech during oral reading and monologs as well as during a rest condition and found that PWS were different to PWDS in both the medial (ITN) and lateral (ETN) systems proposed by Alm (2004). PWS had significantly more activity in the BG (including the left putamen) during an eyes closed rest condition but significantly less activity during speaking conditions. This was thought to result in difficulties in performing fine-grained movement that may extend to speech and explain the fact that other studies observed increased activation of these regions in speech conditions like oral reading and monolog. More specifically though, if it is the case that the BG are overactive during rest and not just underactive during speech, it would indicate abnormalities in stuttering are not solely confined to speech. That is to say, the problem spans a number of domains because there are functional differences in neural activation occurring in the absence of speech.

If abnormalities of the ITN are causally related to stuttering, then it could be expected that effective speech therapy should produce measurable changes in the neural activity of these structures rather than in the areas compensating for stuttering. To this end, Giraud et al. (2008) examined neural activity using fMRI before and after speech therapy in a group of PWS. Therapy consisted of 3 weeks of undergoing an inpatient program focusing on biofeedback for syllable prolongation, soft voice onset and smooth sound transition. The researchers found that activity in the caudate positively correlated with stuttering severity before speech therapy but not after. Since the caudate was positively correlated with severity rather than negatively correlated with it, the speech therapy appeared to target causal rather than compensatory regions.

Similarly, if the ITN is related to stuttering this will not only be reflected in measures of neural activity but also in terms of the connections within the ITN. Lu et al. (2010) used structural equation modeling to compare causal relationships and function in the ITN in PWS and PWDS during a picture-naming task. Although there were no significant differences between stuttering and non-stuttering speakers in the output of the SMA to the BG, there were significant differences between the groups in the output of the BG to the SMA. More specifically, whereas PWDS showed a strong negative projection from the BG to the pre-SMA, PWS showed a positive projection from the BG to the pre-SMA Lu et al. (2010) interpreted their finding of abnormal output of the BG to the SMA as reflecting the difficulties PWS have in updating the timing and sequencing of movement. Interestingly, like Lu et al. (2010), a number of other studies have also shown altered patterns of activity in the SMA in relation to the perception and planning of speech in stuttering (Chang et al., 2009, 2011). Taken together, these findings, are consistent with the notion that stuttering is the result of dysfunctional processes that engage core structures within the proposed ITN: the BG and the SMA.

## Lesion studies of the ITN in PWS

If dysfunction in the ITN is thought to cause stuttering, then it follows that damage to these regions may result in stuttering. When stuttering develops following a lesion to the brain it is known as acquired or neurogenic stuttering (for review see Lundgren et al., 2010). There is evidence that damage to the ITN results in stuttering. For example a recent study by Tani and Sakai (2011) examining five patients with BG lesions (two with bilateral putamen lesions, two patients with bilateral BG lesions and one patient with a left putamenal lesion) but without aphasia, found that they exhibited dysfluencies such as syllable repetitions, part word repetitions and frequent blocks. Importantly, these patients' symptoms mimicked the characteristics of developmental stuttering in that almost all stuttering occurred on the initial syllable of a word. In a number of case studies, Ciabarra et al. (2000) describe a right-handed woman with a left BG lesion, and a woman with a left corona radiata, putamenal and subinsular infarct who both stuttered. Similarly, a number of different case studies have reported the onset of stuttering following damage to the SMA (Alexander et al., 1987; Ackermann et al., 1996; Chung et al., 2004). Furthermore, direct electrical stimulation of the SMA has also been shown to induce stuttering (Penfield and Welch, 1951). These findings are consistent with the notion that damage to the SMA can cause speech disorders and that the SMA is linked with the rhythmic control of speech (Jonas, 1981). This and other works have prompted investigation into the role of the SMA in rhythmic movements of the mouth (MacNeilage and Davis, 2001) as well as dissociations between the pre-SMA and the SMA-proper in rhythmic timing (Schwartze et al., 2012).

## Neuroimaging studies of the ETN system in PWS

There are studies hinting that deficits to the ITN are causing stuttering, but what proof is there that the ETN is recruited to compensate for this? To answer this question, we turn to fMRI studies of PWS. Braun et al. (1997) found the CB to be overactive in PWS during stuttered and fluent speech and it has been suggested that this is a compensatory mechanism for stuttering (see also Alm, 2004). In a meta-analysis of PWS, Brown et al. (2005) identified three neural signatures of stuttering. These neural signatures were the absence of auditory activation bilaterally, the over-activation of the right IFG and the over-activation of the CB. These findings have since been partially replicated by Lu et al. (2010) who found over-activation of the right IFG and the CB (but not the absence of bilateral auditory activation) and interpreted them as compensating for stuttering. Ingham et al. (2012) examined speech during oral reading and monologs as well as rest, finding that PWS exhibited increased cerebellar activity which was negatively associated with stuttering, indicating that the ETN may indeed be compensating for the ITN. A similar study, examined resting state functional connectivity of PWS before and after speech therapy in stuttering and non-stuttering adults (Lu et al., 2012). These authors found increased resting-state-functional-connectivity between the midline CB and a network of regions (comprised of the medial frontal gyrus, the SMA and the left IFG) at rest for PWS relative to PWDS. For the PWS who received intervention as compared to the PWS who did not receive intervention (and PWDS), the resting-state-functional-connectivity in the midline CB returned to normal levels and was correlated with an increase in fluency. As such, Lu et al. (2012) suggested the CB was likely compensating in stuttering. In addition to these, other studies have associated the CB with compensatory activation in PWS (e.g., De Nil et al., 2008; Watkins et al., 2008).

While there is overlap in the neural structures responsible for external timing and compensation for stuttering, it does not automatically follow that the ETN is compensating for deficits in internal timing in PWS. However, there is fMRI evidence showing that the CB and the right IFG specifically compensate for deficits in the BG with respect to timing tasks in those who have Parkinson's Disease (PD). For example, Jahanshahi et al. (2010), investigated the differences in neural activation between PD patients and controls in and the synchronization continuation task. They also examined the effect of administering apomorphine (a non-selective dopamine agonist) on neural activation in the PD patients. Results showed that for healthy controls synchronization and continuation tapping (relative to a control reaction time task) was associated with significantly greater activation in the nucleus accumbens and caudate, a pattern not found in PD patients. In contrast, individuals with PD showed greater activation in the bilateral cerebellar hemispheres, right thalamus and left midbrain during both phases of finger tapping. Administration of apomorphine to the PD patients appeared to normalize activity, both increasing the connectivity between the caudate and putamen and frontal regions as well as decreasing activity in the CB. Thus, the authors suggested that increased cerebellar activation was likely compensating for the impaired functioning of the BG. Sen et al. (2010) found increased cerebellar-thalamo-coritical (CTC) activation as PD progressed, perhaps indicating an increasing need to compensate for loss of function in the striato-thalamo-cortical networks (STC). This increase was only observed during continuation tapping and was not evident during synchronization tapping suggesting that the CTC (i.e., the ETN) was compensating for the STC (i.e., the ITN). The dissociation between the ITN and the ETN may seem problematic given both the CB (part of the ETN) and the SMA (part of the ITN) are thought to compensate for deficits in the BG during self initiated hand movements in the early stages of PD (Eckert et al., 2006). Nevertheless, this could suggest that part of the ITN (the SMA) may still be able to compensate for deficits in other parts of the ITN (the BG) when degeneration is not particularly severe.

## Compensation by the right IFG in stuttering

An increasing number of studies have reported anomalous activation of the right IFG in a variety of speech tasks (e.g., Fox et al., 1996; Brown et al., 2005; Sowman et al., 2012) in PWS. Several studies found that increases in right IFG activation during overt reading (Preibisch et al., 2003; Lu et al., 2010) that were positively correlated with speech fluency in PWS and thought to be a non-specific compensatory mechanism because the activation was not specifically related to speech production. Examining the effect of external auditory pacing on the speech of PWS Toyomura et al. (2011) found that, relative to a PWDS, the PWS showed more activation in the right IFG (along with bilateral auditory cortices) during both choral speaking and when speaking in time with an isochronous metronome. There are also reports of increased right frontal connections in adults who began stuttering as children (i.e., developmental stuttering) relative to adults who began stuttering later in life following a psychological trigger and without evidence of brain injury (Chang et al., 2010). This evidence suggests that the longer a PWS has been compensating for their stuttering, the greater the activity in the right IFG.

It is worth noting that Goldberg's formulation of the lateral system (upon which the ETN partially maps) does not contain the right IFG. Why then should right IFG be considered a part of an ETN that compensates for a dysfunctional ITN in stuttering? This question is particularly relevant when considering that the simplest explanation for right IFG involvement in stuttering is that it compensates for deficits in the left IFG (see Kell et al., 2009). Kell et al. (2009) associate the left IFG with processing of rhythm and sensorimotor feedback and it is possible that the right IFG may perform a similar function. Recently, the right IFG has been recognized as part of a “core timing network” (Wiener et al., 2010) that is recognized to be strongly connected both functionally and structurally to the ITN (Kung et al., 2013; Brittain and Brown, 2014). In particular, the right IFG may only become active when a task is more demanding. That is to say, the difficulty of compensating for deficits in internal timing by external timing regions might account for why there was over-activation of only the CB during speech, but not the right IFG during rest in PWS (Lu et al., 2012). A second, though not mutually exclusive explanation is that while the CB is able to compensate for timing deficits, its ability to do so is limited. This is evident in the case of individuals with PD where behavioral performance worsened despite increases in compensatory activation in the CB (Sen et al., 2010). A similarly limited ability of the cerebellar systems to compensate for deficits in timing may be occurring in PWS as evidenced by the reduced integrity of cerebellar tracts in both the left and the right hemispheres (Connally et al., 2013). Since the ETN has a limited capacity to compensate for deficits in the ITN, the assistance of the right IFG may be required to maintain normal timing functions. A third possible explanation is that the model proposed by Goldberg (1985) (where the ETN is comprised of the CB and the PMC) is incomplete and requires the addition of the right IFG as a secondary part of the system. Importantly, the right IFG is not likely to be the only region that is be compensating for stuttering. There are many other regions like the orbitofrontal cortex that could found to be compensating depending on the task and motor regions involved (see Kell et al., 2009; Sowman et al., 2012). Our contention is that the right IFG forms part of a network that compensates for deficient internal timing.

## Behavioral studies of timing in PWS

If stuttering is the result of dysfunction in the ITN, and the ITN is important for timing, then it follows that PWS should exhibit deficits in behavioral performance on timing tasks. To this end several groups have found significant differences in asynchrony and variability of tapping between PWS and PWDS. For example, measuring the timing variability of reading sentences or nursery rhymes or tapping, Cooper and Allen (1977) found that PWS were consistently more variable in the length of time it took them to read sentences, paragraphs or nursery rhymes, and in their inter-tap intervals compared to PWDS. Brown et al. (1990) found that PWS were slower and less variable than PWDS at repeating the phrase “ah” and tapping their fingers as at their own pace compared to PWDS, findings they interpreted to represent less flexible timing systems which were more susceptible to breakdown. Similarly, when examining the timing intensity and variability of externally timed speech, Boutsen et al. (2000) showed that although both PWS and PWDS exhibited similar intensities when producing syllables, PWS were significantly more variable in their inter-onset vocalization times (analogous to the inter tap interval in tapping tasks). Additionally, Zelaznik et al. (1997) found that PWS were more variable on bimanual finger tapping (something more demanding than unimanual finger tapping) relative to PWDS. Similarly, Hulstijn et al. (1992) found that on a task which required the coordination of finger tapping and vocal responses (tapping in time with vocalizing the word “pip”), PWS exhibited greater variability than PWDS. More recently, Olander et al. (2010) compared hand-clapping variability in CWS and CWDS. While there was no difference in mean clapping rate, there were significant differences between groups in the variability of the clapping rate. This variability was bimodally distributed, with 60% of CWS showing variability that was greater than the worst performing CWDS. The remaining CWS showed variability in clapping that overlapped with that of the CWDS. Interestingly, this number approximately corresponded to the number of children that spontaneously recover and whose stuttering persists. As a result, the authors suggested that the motor timing deficit may be predictive of recovery from stuttering. Later, Foundas et al. (2013) found that when male CWS were required to tap as fast as possible in a given time period, most were better when tapping with their left rather than right hands as compared to most male CWDS who showed an advantage for their right hand. A recent behavioral study has found robust differences in tapping performance between CWS who stutter compared to CWDS (Falk et al., 2014). In contrast to the CWDS, the CWS not only tapped earlier and were less consistent in tapping, but also failed to improve with age.

However, a number of studies have compared the asynchrony and variability of PWS and PWDS on externally or internally timed vocal or oral motor movements and found similar levels of variance between the groups (e.g., Hulstijn et al., 1992; Melvine et al., 1995). Similar results have been obtained by Zelaznik et al. (1994) who compared PWS and PWDS on externally and internally timed manual responses for isochronous intervals and found that the groups did not differ in behavioral performance. Likewise, Max and Yudman (2003) found PWS and PWDS displayed highly similar levels of asynchrony and variability for finger tapping and producing vocalizations for multiple isochronous intervals. Overall, the behavioral studies investigating the timing abilities of PWS have produced mixed results. While some studies have found differences between PWS and PWDS, many have failed to find differences between groups. From this research, it might seem appropriate to conclude that stuttering is not a disorder of timing and that the links between stuttering and deficits in production of timed limb movements is tenuous at best. One possible explanation is that motor control of limbs and speech is different both centrally and peripherally (Kent, 2000). However if this were indeed the case, then it would be hard to explain why some studies did find significant differences between PWS and PWDS in non-speech motor tasks. Moreover, there is evidence of common timing systems across modalities (Franz et al., 1992) and it has been stressed that the behavioral differences between PWS and PWDS are not confined to the speech production system and instead appear to be generalized deficits (Max et al., 2003). There are other possible explanations for the failure to find behavioral differences between groups which can, in part, be attributed to compensatory neural activity and task difficulty.

## Tentative suggestions for timing deficits in PWS

The substantial number of studies finding no difference in timing behavior in PWS and PWDS is inconsistent with the notion that stuttering could be considered a disorder of timing. How then can we resolve these seemingly paradoxical findings with the consistent observation that neural regions involved in internal timing display anomalous function and structure in stuttering? The absence of a difference at a behavioral level does not imply the absence of differences at a *neural* level. Even a task as simple as tapping a finger or vocalizing to a metronome recruits a complex network of brain regions each with a variety of different functions (Repp and Su, 2013). Moreover, there may be differences at the neural level in the absence of differences at the behavioral level precisely because PWS are compensating for deficits in internal timing. Such a possibility is highlighted by the findings of Neef et al. (2011), who, utilizing inhibitory TMS, showed PWS did not exhibit behavioral differences in timing prior to stimulation but did exhibit behavioral differences subsequent to stimulation. If the suggestion that PWS demonstrate similar behavioral performance as a result of re-organization is plausible, then PWS should exhibit compensatory neural activity in regions associated with external timing of movement that are specifically compensating for deficits in the internal timing of movements. This indeed appears to be the case as both the CB and the right IFG seem to be compensatory regions in stuttering; both appear to be associated with timing, and both may specifically be compensating for deficits in the BG's control of timing tasks. Although speculative, this strongly suggests that the compensatory response to stuttering that occurs during speech is occurring as a result of deficits in the ITN. It perhaps explains why, in some studies at least, PWS have not shown differences in asynchrony (the difference in time between taps and the pacing signal) or variability (in the time between taps) on tapping tasks compared to PWDS. However, any failure to find a difference between these groups may also be attributed to task related effects such as the motoric or temporal complexity.

Many of the behavioral studies investigating timing abilities in PWS employed simple motoric and temporal tasks. Tapping at isochronous intervals is, as a task, relatively easy and this ease may explain a lack of differences in behavioral performance between PWS and PWDS, a problem that may extend to differences in regional brain activation in neuroimaging studies. Imaging data from early research on finger movements shows that the amount of cerebral blood flow to a particular region depends upon the complexity of the task (Shibasaki et al., 1993). Simple tasks are, *ipso facto*, not sufficiently motorically demanding to engage parts of the brain normally employed in more complex tapping tasks and which are impaired in PWS. This principle has been demonstrated experimentally in a number of studies. For example, Zelaznik et al. (1994) failed to find behavioral differences when comparing unimanual tapping performance, but successfully found differences in the same group of stuttering participants when examining bimanual tapping at an isochronous interval (Zelaznik et al., 1997). Similarly, increasing the syntactic complexity of words surrounding a to-be-repeated phrase, decreased speech motor stability for PWS as compared to PWDS (Kleinow and Smith, 2000).

In the same way that increasing the difficulty of the motor movement associated with the task could better reveal differences (should they exist) in behavioral performance and neural activation, so too could placing more strain on the systems governing temporal control of movements. Whereas Webster (1985) failed to find a difference in behavioral performance for PWS during bimanual tapping in a 1:1 ratio (that is one tap of the right hand for every tap of the left hand), Webster (1990) found that PWS took a substantially longer time to tap the required number of times when tapping in a ratio of 2:1 (that is two taps of the left hand for each tap of the right hand) than PWDS. Tapping at an uneven ratio (2:1) places significantly more demand on the neural systems governing timing than does tapping in an even ratio (1:1). This suggests that PWS are much less efficient in coordinating motor output to complex temporal patterns. Similarly, Lewis et al. (2004) demonstrated that parametrically increasing the number of different intervals in a series of tones resulted in a corresponding increase in neural activation in regions associated with timing. These studies show that, increasing the demands on temporal processing is more likely to yield differences in behavior and by extension, in neural activation. This is particularly relevant in the case of speech since speech is rarely perfectly isochronous but rather quasi-periodic (Martin, 1972). Speech contains multiple levels of temporal complexity (Kotz and Schwartze, 2010; Goswami and Leong, 2013) and is therefore substantially more demanding than tapping at an isochronous interval or in a 1:1 ratio. That is to say, differences in the complexity of rhythms required for speech and finger tapping may explain why most timed movements are relatively normal in PWS. Additionally, the timing required for speech control is robust to interference so difficulties in timing movements or speech may only become evident under increased cognitive loads (e.g., Saltuklaroglu et al., 2009). If PWS were compared to PWDS on a tapping task that contained a similar degree of temporal complexity usually required by speech, then clinically meaningful differences in behavior are likely to emerge. While there is a theoretical distinction between motor and temporal complexity, in practice, this distinction may not be so clear. Using near infrared spectroscopy (a means to measure the level of deoxygenated blood from the scalp somewhat analogous to how fMRI measures neural activity) Koenraadt et al. (2013) found that that the two may not be mutually exclusive. Tapping at multiple frequencies activated larger portions of the motor cortex than tapping at single frequencies. The extent to which manipulating motoric and temporal complexity are able to elicit behavioral differences in timing between PWS and PWDS remains to be tested by future research. Yet, even if these tasks are unable to elicit such differences in PWS, future research investigating the overlap between stuttering and timing should consider the use of neuroimaging techniques.

## Directions for future research

There appears to be a vast gap in the stuttering literature particularly with respect to neuroimaging and brain stimulation of timing tasks. In particular, we know of no fMRI or positron emission tomography (PET) studies that specifically examined internally or externally timed movements in PWS using either simple or complex temporal intervals despite the long theoretical history of an association between deficient timing and stuttering. The timing deficits we propose to exist in PWS are only tentative suggestions and remain to be verified by future research. Our proposal can nevertheless be used to generate a number of testable hypotheses. For example, it could be hypothesized that PWS show impaired behavioral performance and corresponding neural activation in tasks that require the internal timing of movements (the continuation phase of a finger tapping task) as opposed to the external timing of movements (the synchronization phase of a finger tapping task).

Likewise to the best of our knowledge, there are no studies investigating neural oscillations in PWS in response to isochronous or non-isochronous tones either by passive listening, finger tapping or vocalizations. Given the role of neural oscillations in timing (Arnal, 2012), it would be interesting to investigate how they might differ between PWS and PWDS in the context of a timing task. With respect to studies of brain stimulation, no studies have yet examined the effect of distuptive TMS on the right IFG, the SMA or the CB in PWS in a timing task. Although speculative, it might be expected that tapping in time to a metronome (external timing) will be relatively unimpaired because PWS can rely on the CB and premotor cortices much in the same way as non-stuttering adults do. However for self-paced tapping it might be expected that following inhibitory TMS to the right IFG, PWS will be significantly impaired because they cannot rely on either the right IFG or the BG. In contrast, PWDS will be able to rely on the BG, but not the right IFG. The compensatory function of the right IFG in stuttering is biologically plausible in that it forms part of a core timing-network (Wiener et al., 2010), is functionally interconnected with the BG (Kung et al., 2013) and is utilized for the processing of speech rhythm (Geiser et al., 2012).

While this article focused on the neural correlates of the ITN and the ETN during the perception and production of rhythmic movements and stimuli, there are many other tasks that probe these networks. The finger-tapping task is a continuous task that is often conducted in the presence of a regular external stimulus. It is possible that the regular external stimulus reduces behavioral variability and (possibly the associated) neural activity much in the same way that it is able to temporarily induce fluency in PWS. It would therefore be prudent to examine the timing abilities of PWS on tasks that do not contain such regular stimuli or where there is a disruption to the external stimuli. In line with the hypothesis of impaired internal timing and the hypothesized compensatory increases in regions associated with the processing of external timing of movements, it might be expected that PWS are more reliant on external cues. As such it would be interesting to test abilities of PWS to judge whether a “test interval” is longer or shorter than a “reference interval” and how these judgments are influenced by the presence of a “distractor interval” that they must ignore (see Rao et al., 2001). To this end, we know of no studies that have examined temporal judgment deficits in PWS either behaviorally or neurologically. More generally, if it is demonstrated that PWS exhibit deficits in timing, it would be particularly interesting to see if there is any dissociation between these different types of timing tasks or modalities; There may for example, be a dissociation between motor timing or judgment duration or between auditory and visual timing.

## Concluding remarks

In conclusion, we provide a theoretical framework with which to view stuttering as a disorder of timing. This paper reviews converging evidence from neuroimaging and brain stimulation experiments showing a great degree of overlap between the structures engaged in the internal timing of movements and the regions thought to be causally involved in stuttering. We also provide evidence of overlap between the neural structures engaged in the external timing of movement and link them with compensatory activity in PWS. We further highlight significant gaps in the literature and suggest avenues for further research motivated by this overarching theory. More generally, this article highlights anomalies in the functional activations and the structural anatomy of the areas involved in the processing of time in stuttering, that are linked to the dysfluent production of speech and should motivate further research in the field.

### Conflict of interest statement

The authors declare that the research was conducted in the absence of any commercial or financial relationships that could be construed as a potential conflict of interest.

## Abbreviations

BG: Basal ganglia
CB: Cerebellum
CTC: Cerebellar-thalamo-cortical
CWDS: Children who do not stutter
CWS: Children who stutter
ETN: External timing network
fMRI: Functional magnetic resonance imaging
IFG: Inferior frontal gyrus
ITN: Internal timing network
MEG: Magnetoencephalography
PD: Parkinson's disease
PET: Positron emission tomography
PMC: Premotor cortex
PWDS: People who do not stutter
PWS: People who stutter
SMA: Supplementary motor area
STC: Striato-thalamo-cortical
STG: Superior temporal gyrus
TMS: Transcranial magnetic stimulation
VBM: Voxel based morphometry.
